# Generation of bovine iPSCs from fetal fibroblasts for in vitro myogenesis and cultured meat

**DOI:** 10.3389/fnut.2025.1562981

**Published:** 2025-05-16

**Authors:** Kaiana Recchia, Methi Wathikthinnakon, Fabiana Fernandes Bressan, Kristine Freude

**Affiliations:** ^1^Surgery Department, Faculty of Veterinary Medicine and Animal Sciences, University of São Paulo, São Paulo, Brazil; ^2^Department of Veterinary and Animal Sciences, Faculty of Health and Medical Sciences, University of Copenhagen, Frederiksberg, Denmark; ^3^Department of Veterinary Medicine, Faculty of Animal Sciences and Food Engineering, University of São Paulo, Pirassununga, SP, Brazil

**Keywords:** bovine, episomal reprogramming, lentiviral reprogramming, pluripotent stem cell, *in vitro* myogenesis, myotube, cultured meat

## Abstract

**Introduction:**

Emerging biotechnologies are increasingly being explored for food production, including the development of cell-cultivated meat. Conventional approaches typically rely on satellite cell (SC) biopsies, which present challenges in scalability. Bovine induced pluripotent stem cells (biPSCs) represent a promising alternative due to their capacity for self-renewal and developmental plasticity.

**Methods:**

This study utilized both lentiviral (integrating) and episomal (non-integrating) reprogramming strategies to generate biPSCs suitable for myogenic differentiation. Bovine fetal fibroblasts (bFFs) were reprogrammed using episomal vectors pMaster K and pCXB-EBNA1, leading to the emergence of putative iPSC colonies 13 days post-nucleofection. A clonal line, bFF-iPSCs pMK, was selected for further analysis.

**Results:**

The bFF-iPSCs pMK line expressed key pluripotency markers including alkaline phosphatase (AP), *OCT4*, *SOX2*, and *NANOG*, and was stably maintained for over 33 passages, although episomal plasmids remained detectable. *in vitro* myogenic differentiation was assessed by comparing this line to a previously established lentiviral reprogrammed line (bFF-iPSCs mOSKM). Both lines exhibited downregulation of pluripotency markers and upregulation of the early myogenic marker *PAX3*. By day 30, the bFF-iPSCs pMK line formed elongated, multinucleated cells characteristic of myotubes and displayed a corresponding gene expression profile.

**Discussion:**

These results provide new insights into bovine *in vitro* myogenesis and its application in cultured meat production. While promising, the study also highlights the difficulty in achieving complete myogenic differentiation, indicating a need for further optimization of differentiation protocols.

## Graphical abstract

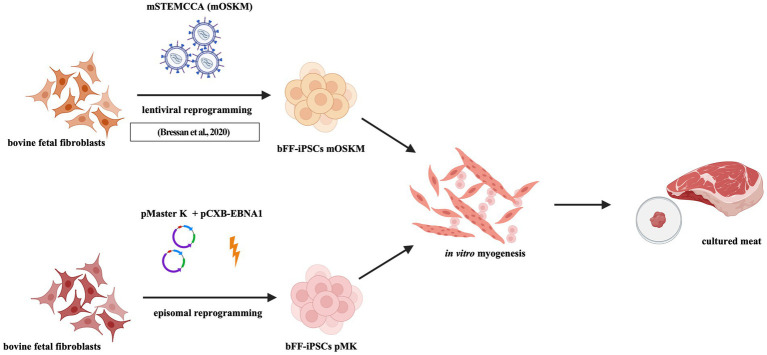

## Highlights

•Bovine fetal fibroblasts were reprogrammed into pluripotency using episomal or lentiviral methods, and biPSCs were analyzed regarding myogenic differentiation potential.

## 1 Introduction

The advent of induced pluripotent stem cells (iPSCs) has significantly advanced the study of *in vitro* myogenesis (1–4). The ability to generate muscle cells *in vitro* is pivotal for alternative protein production, particularly as global demand for animal-derived products rises with population growth. As a result, there is a growing need for innovative solutions in the food system to revise current livestock systems or to produce animal proteins while minimizing environmental impact and mitigating the challenges of intensive animal farming (5, 6). In this context, *in vitro* technologies, including cultured meat (CM), also known as cell-based food, are presented as a promising alternative to traditional meat production (7–10).

One critical step in cultured meat production, among others, is the isolation and efficient differentiation of SCs into mature muscle fibers (10, 11). This differentiation depends on the timely activation of key lineage markers, such as MSGN1 and TBX6 in presomitic mesoderm, followed by expression of PAX3 and PAX7 in skeletal muscle progenitors (SCs), which subsequently differentiate into myoblasts through MYF5 and MYOD1 expression (12). However, challenges remain in CM production due to scalability issues, nutritional optimization, sensory properties, and consumer acceptance, despite significant investment in addressing these hurdles (13, 14, 64, 65). Current SC cultures have limited expansion potential, often requiring multiple tissue collections for large-scale production.

iPSC technology offers a solution by enabling the generation of large quantities of myogenic progenitors without the need for tissue sampling (3, 9, 15–18). The plasticity of iPSCs allows for *in vitro* myogenesis, as demonstrated in human models (1–3, 15, 16, 19), and muscle cell generation from human iPSCs has already been achieved within a month of differentiation, enabling large-scale production of myogenic progenitor cells (1, 15, 18).

Bovine iPSCs have also shown promise for generating myoblasts and myogenic progenitors. Although bovine embryonic stem cells (ESCs) have been used in short-term cultures to derive muscle cells (20, 21), iPSCs offer distinct advantages. They can be derived from abundant and easily accessible tissues, such as skin or blood, overcoming the limitations associated with ESCs, which require embryos that are more difficult to obtain and are often limited in supply (17). Induced reprogramming has been successfully performed in bovine cells, predominantly using lentiviral vectors (22–28) and lentiviral reprogramming methodology; nevertheless, no episomal protocol and no episomal reprogramming protocols have been reported for this species to date. The application of iPSC-derived myogenesis in large animal models, such as bovines, has the potential to advance alternative protein production technologies.

This study aims to generate SCs and myotubes from bovine iPSCs, providing novel insights into the myogenic differentiation process. The results not only enhance the understanding of myogenesis but also address critical challenges in cell culture techniques and alternative protein production.

## 2 Materials and methods

### 2.1 Bovine fetal fibroblasts

The bovine fetal fibroblasts (bFFs) were previously isolated and characterized (29). The cells were cultured in bFFs media, which was composed of DMEM/F12 (Gibco, cat. 31,331–028) supplemented with 10% FBS and 10 U/mL penicillin and streptomycin at 5% CO_2_ and 38.5°C.

### 2.2 Lentiviral-derived biPSCs

The bFF-iPSCs reprogrammed with a lentivirus containing the murine Stem Cell Cassette (STEMCCA) harboring the murine vesions of Oct4, Sox2, Klf4 and c-myc (mOSKM) was previously generated and characterized (23). The cells were cultured with KnockOut™ Dulbecco’s Modified Eagle Medium/Nutrient Mixture F-12 (KO DMEM/F12 from Gibco, cat. 12660012) supplemented with 20% KnockOut™ Serum Replacement (KSR from Gibco, cat. 10828028), 10 U/mL penicillin and streptomycin (Gibco, cat.15140122), 0.1 mM B-Mercaptoethanol (Sigma, cat. 31350-010), 1% Non-Essential Amino Acids (NEAA), 1% GlutaMAX (Gibco, cat. 35050061) and 10 ng/µl (bFGF from Peprotech, cat. 100-18B-250UG).

### 2.3 Episomal reprogramming and biPSCs culture

For cellular reprogramming of bovine fetal fibroblasts (bFFs) using episomal plasmids, 1×10^6^ bFFs were nucleofected with 3 μg pMaster K (30) (Addgene, cat. 165,081) in association with 1 μg pCXB-EBNA1 (31) (Addgene, cat. 41,857) using the Nucleofector 4D Lonza and the P3 Primary Cell Solution Box (Lonza, cat. PBP3-02250), program CA-137. After the nucleofection, the cells were divided into two wells of six-well plates previously coated with Matrigel (Corning, cat. 354,277) and cultured with bFFs media.

After 3 days, the media was changed to bovine pluripotent stem media: mTeSR™ 1 Basal Medium (Stem Cell Technologies, cat. 85,850) supplemented with 10 U/mL penicillin–streptomycin (Gibco, cat.15140122), 0.1 mM B-Mercaptoethanol (Sigma, cat. 31,350–010), 28.94 μg/mL ascorbic acid (Sigma, cat. A8960), 1 μM CHIR99021 (Sigma, cat. SML 1046), 20 ng/mL activin A (R&D system, cat. 11,348-AC), 5 μM XAV939 (Sellect Chem, cat. X3004), 0.3 μM WH-4-023 (SelleckChem, cat. S7565), and 10 ng/mL human FGF-basic. The media was changed every other day for up to 30 days at 5% CO_2_ and 38.5°C conditions. Once colonies were observed, they were manually replated on top of a monolayer of Mitomycin C-treated murine embryonic fibroblasts (MEFs, Sigma-Aldrich, cat. M4287). The media was changed daily, and the cells were cultured in 5% de CO_2_ at 38.5°C.

### 2.4 biPSCs differentiation into satellite cells—*in vitro* myogenesis

biPSCs were subjected to *in vitro* myogenesis differentiation using the commercially available Skeletal Muscle Induction Medium (ASMBIO, cat. SKM01), following the recommended protocol, previously validated for human PSCs differentiation (3, 19, 32). First, the biPSCs were dissociated into single cells and plated on six-well plates previously coated with Matrigel (Corning, cat. 354,277) and cultured with SKM01 media for 6 days. Then, the cells were snap-frozen and analyzed via Reverse Transcription quantitative Polymerase Chain Reaction (RT-qPCR). The media was changed every other day, and cells were cultured in 5% de CO_2_ at 38.5°C.

The bFF-iPSC pMK at passage 33 was submitted to an adapted protocol previously described for human iPSCs (1, 2, 15). In brief, the cells were pre-differentiated following the methodology I with SKM01 on Matrigel until day 8, after which the media was changed to DK-HIFL composed of DMEM media supplemented with 2 ng/mL insulin-like growth factor 1 (IGF-1, Peprotech, cat. 100–11), 10 ng/mL hepatocyte growth factor (HGF, Peprotech, cat. 315–23), 20 ng/mL bFGF (Peprotech), and 0.5 μM LDN193189 (Sigma, cat. SML0559). The media was changed daily until D10, when the cells were cultured in DK-I media (DMEM, 15% KSR (Gibco) supplemented with 2 ng/mL IGF1). After D12, the cells were cultured with DK-HI (DMEM/F12, 15% KSR, 2 ng/mL IGF1 and 10 ng/mL HGF) until D30. The media was changed every other day, and the cells were cultured in 5% de CO_2_ at 38.5°C.

### 2.5 Characterization of biPSCs and satellite cells

#### 2.5.1 Alkaline phosphatase and immunocytochemistry of pluripotency markers

The detection of AP was performed with the Leukocyte Alkaline Phosphatase kit (Sigma, cat. 86R) following the manufacturer’s instructions and reported in different iPSC species (23, 33, 34). Immunocytochemistry (ICC) for pluripotency markers was performed via fixation of biPSCs with 4% Paraformaldehyde for 15 min, followed by permeabilization and blocking with 0.5% Triton-X and 5% Donkey Serum for 1 h at room temperature. Afterward, biPSCs were rinsed three times with 0.05% Triton-X in Phosphate-Buffered Saline (PBS) for 5 min (wash solution) followed by incubation with the primary antibodies OCT3/4 (1:200, Santa Cruz, SC-5279, Mouse), SOX2 (1:200, R&D, cat. AF2018, Goat), and NANOG (1:200, R&D, cat. AF1997, Goat) overnight at 4°C. The following day, biPSCs were rinsed in the wash solution and incubated with the respective secondary antibodies: donkey anti-Mouse (1:500, Alexa Fluor 594, Invitrogen, cat. A21203) or donkey anti-Goat (1:500, Alexa Fluor 594, Invitrogen, cat. A11058). The cell nuclei were labeled with DAPI (1:1000, ThermoScientific, cat. 62,248) and analyzed using the EVOS™ cell imaging system.

#### 2.5.2 Plasmid detection analyses—genomic PCR and electrophoresis

DNA from bFFs-iPSC colonies generated through episomal reprogramming (pMaster K) was extracted using the DNeasy Blood & Tissue kit (QIAGEN, cat. 69,506), following the manufacturer’s instructions. DNA was analyzed and quantified using a spectrophotometer (NanoDrop Lite Plus Spectrophotometer, ThermoScientific, cat. NDLPLUSPRGL). To test for episomal plasmid detection, a PCR was performed using primers designed to amplify the oriP region (Supplementary Table 1) (30). For the reaction, 1 μL primer mix (forward and reverse at 100 uM), 7 μL SYBR Gold (Invitrogen, cat. S11494), 5 μL Nuclease-Free, water (Sigma, cat. WH502), and 100 ng/ul sample DNA were mixed. The amplification program was run in a thermocycler at 94°C for 10 min; 40 cycles at 94°C for 30 s, 60°C for 30 s, 72°C for 1 min, and a final extension at 72°C for 7 min. The PCR product was run in an electrophoresis gel composed of 1% TAE buffer (Thermo Scientific, cat. B49) and 2% UltraPure™ Agarose (Invitrogen, cat. 16,500,500), running at 100 mV for 40 min. The band was evaluated by photodocumentation.

#### 2.5.3 Transcript quantification—RT-qPCR

RNA extraction was performed with the RNeasy Plus Mini kit (QIAGEN, cat. 74,134) following the manufacturer’s guide. The Ribonucleic Acid (RNA) samples were analyzed regarding quantity and quality using a spectrophotometer (NanoDrop Lite Plus Spectrophotometer, cat. NDLPLUSULCA). Reverse transcription of the extracted RNA was performed using the commercial GoScript™ Reverse Transcriptase (Promega, cat. A2801), according to the manufacturer’s instructions.

The target genes for the evaluation of pluripotency via RT-qPCR in biPSCs were endogenous *OCT4*, *SOX2*, and *NANOG* due to their importance in the reprogramming and maintenance of pluripotency, and *PAX3*, *PAX7,* and *MYOG* for differentiated cells due to their importance in the differentiation process from early mesoderm into striated myotubes and fibers. The *GAPDH* and *PPIA* genes were used as reference genes (ST1). PCR conditions for amplification were: 95°C for 15 min; 40 cycles of 95°C for 15 s, 60°C for 5 s, and 72°C for 30 s; and 72°C for 2 min; the melting curve was analyzed up to 90 cycles starting at 50°C with a 0.5°C increase. The samples were analyzed and compared based on the relative abundance of transcripts. The PowerUP SYBR Green reagent (Applied Biosystems, cat. A25778) was used in the reactions, and the primers were at 100 nM in standard thermocycling conditions (annealing temperature of 60°C for 45 cycles). Based on duplicates or triplicates, the relative abundance quantification was evaluated using the 2-ΔCT method.

#### 2.5.4 Statistical analysis

Data obtained from the experimental procedures were analyzed using GraphPad Prism version 9.5.0 for Mac (GraphPad Software, San Diego, California United States).^1^ The analyses were performed using technical duplicates or triplicates (*n* = 2 or *n* = 3) for both bFF-iPSCs pMK episomal-derived and bFF-iPSCs mOSKM lentiviral-derived lines. The data were analyzed by one-way ANOVA followed by Tukey’s multiple comparison test or Welch’s *t*-test. A significance level of 5% (*p* < 0.05) was considered for all statistical analyses.

## 3 Results

### 3.1 Bovine fetal fibroblasts iPSCs reprogramming

**Figure 1:**
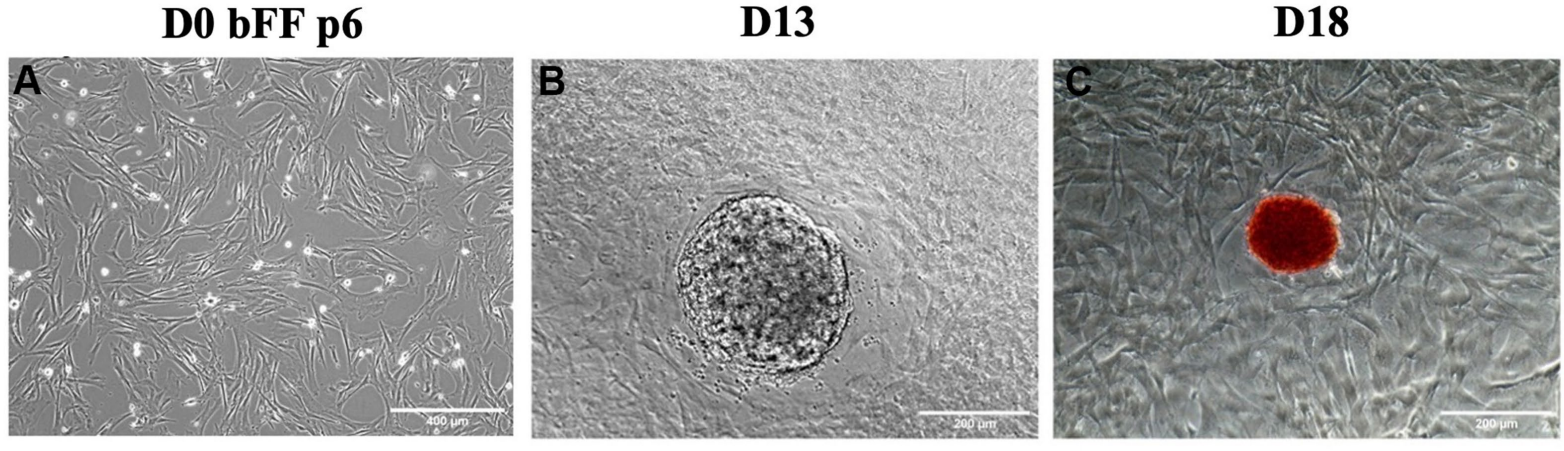
Morphological changes during episomal reprogramming of bFFs. **(A)** Day 0 (D0) of reprogramming protocol in bFFs at passage 6. **(B)** A representative colony was observed on day 13 (D13) after bFFs underwent nucleofection with pMaster K and EBNA1. **(C)** A representative AP-stained colony on day 18 (D18).

bFFs in passage 6 underwent nucleofection with the plasmids pMaster K, which was associated with pCXB-EBNA1. Colony formation was observed after 13 days, and alkaline phosphatase (AP) activity was detected on day 18 when the last manual colony was replated (Figure 1).

**Figure 2:**
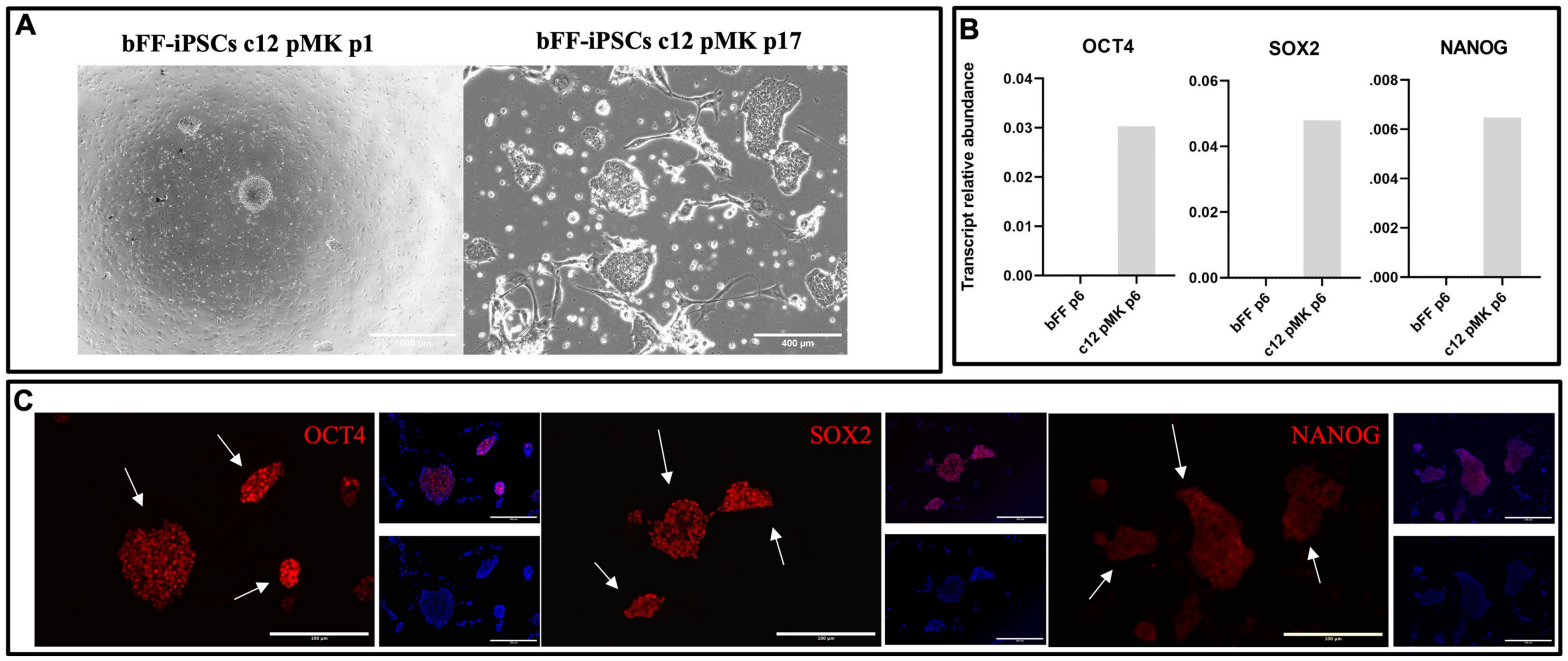
Characterization of clonal line bFF-iPSCs c12 pMK. **(A)** Clonal line bFF-iPSCs c12 pMK after manual picking at passage 1 and cultured until passage 17, displaying typical iPSCs morphology, with limited border and dome-shape appearance (Scale bar: 1000—bFF-iPSCs pMK c12 p1 or 400 μm—bFF-iPSCs pMK c12 p17). **(B)** A relative abundance of endogenous pluripotency transcripts (*OCT4*, *SOX2*, and *NANOG*) was detected in the clonal bFF-iPSCs c12 line at passage 6 but was absent in bFF at passage 6. **(C)** Immunocytochemistry (ICC) analysis confirmed the presence of pluripotency markers OCT4, SOX2, and NANOG in the clonal bFF-iPSCs pMK c12 line at passage 14 (Scale bar: 200 μm).

Fourteen colonies were manually selected and replated onto a monolayer of MEFs. The clonal line 12 was chosen based on its typical iPSC morphology, such as limited borders, dome-shape appearance, and faster proliferation, as well as the presence of transcripts and markers related to pluripotency. Pluripotency markers OCT4, SOX2, and NANOG were detected both at transcript and protein levels, indicating that the biPSCs had been successfully reprogrammed (Figure 2).

Based on these confirmed iPSC characteristics, clonal line 12 (bFF-iPSCs pMK) was selected for subsequent differentiation into satellite cells even though the presence of episomal plasmids was still detectable at passages 12 and 17 (Supplementary Figure 1).

### 3.2 Pluripotency profile between biPSCs generated from different cell lines and protocols

**Figure 3:**
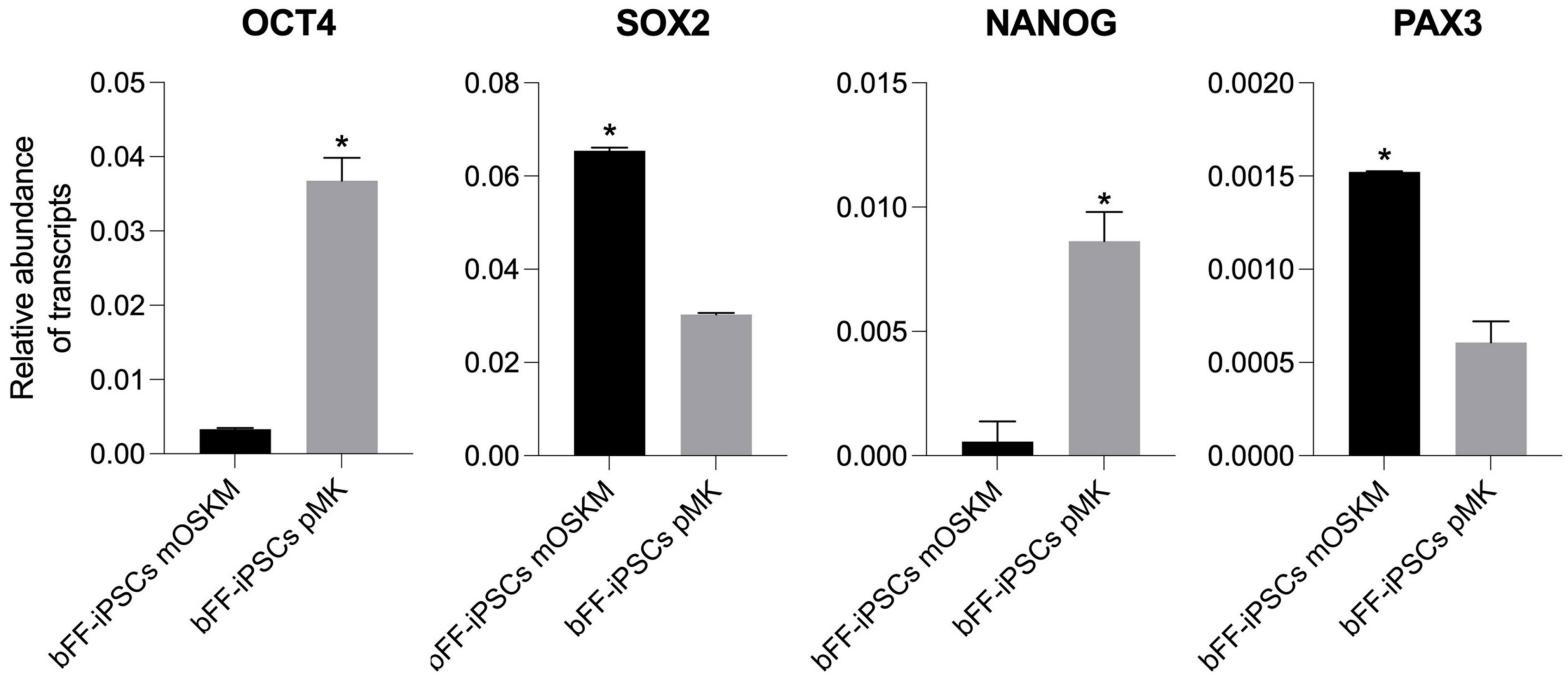
Comparison of pluripotency gene expression and early myogenic marker in bFF-iPSCs. Relative transcript abundance of pluripotency genes OCT4, SOX2, and NANOG, and the early myogenic marker PAX3, in two biPSC lines (bFF-iPSCs mOSKM and bFFs-iPSCs pMK). Endogenous OCT4 and NANOG were more abundant in bFF-iPSCs pMK, while SOX2 levels were comparable between the two iPSC lines. PAX3 expression was higher in bFF-iPSCs mOSKM. Experiments were conducted in technical duplicate, and results are presented as the mean ± standard deviation (SD) to reflect variability. Bars labeled with an asterisk indicate statistically significant differences between groups (*p* < 0.05).

The relative abundance of endogenous pluripotency transcripts and the early muscle progenitor marker *PAX3* was analyzed in both biPSC lines used for the myogenesis differentiation protocol (bFF-iPSCs mOSKM p22 and bFF-iPSCs pMK p5) (35, 36). *PAX3* expression is upregulated during myogenic differentiation of iPSCs, serving as a key marker for early muscle progenitor cells. Its increased expression plays a crucial role in the transition from pluripotency to the myogenic lineage, promoting the proliferation and differentiation of muscle precursor cells (3, 15, 37). Prior to starting the differentiation, both biPSC lines were assessed for the expression of pluripotency markers *OCT4*, *SOX2*, and *NANOG* and the early myogenic marker *PAX3* (Figure 3). All experiments were performed in technical replicates (*n* = 2).

### 3.3 biPSCs and myogenesis differentiation

The myogenesis differentiation of both biPSC lines was performed simultaneously. When differences in the results were observed, they were analyzed and discussed separately in this manuscript. Bovine primary satellite cells, donated by Aarhus University, were used as a control to evaluate the differentiation potential of the biPSCs.

#### 3.3.1 Differentiation of bFF-iPSCs derived from mOSKM lentiviral reprogramming into SCs

**Figure 4:**
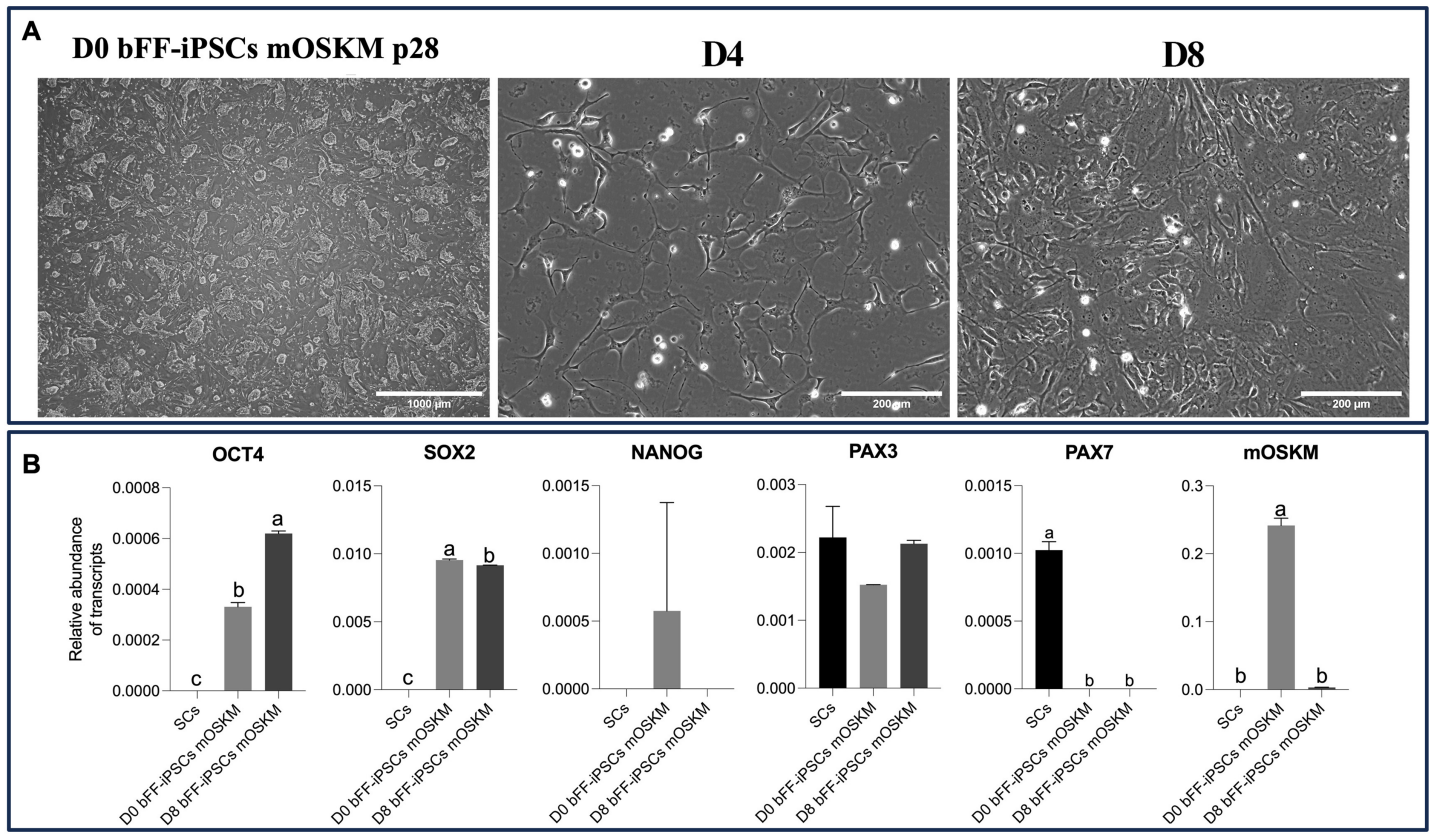
Characterization of biPSC differentiation capacity into the myogenic lineage. **(A)** Morphological changes of bFF-iPSCs mOSKM at passage 28 after 8 days of culture in SKM01 media (scale bar: 1000 μm at D0; 200 μm at D4, D8). By day 4, no colonies were observed, and cells began to elongate, forming small clusters. By day 8, an increased number of elongated, fibroblast-like cells were evident. **(B)** Relative transcript abundance of pluripotency and myogenesis-related genes in biPSCs, differentiated cells, and bovine primary satellite cells. Pluripotency markers (*OCT4*, *SOX2*, and *NANOG*) were highly expressed in D0 bFF-iPSCs mOSKM, with levels decreasing following differentiation. The myogenic marker *PAX3* was more abundant in bovine primary satellite cells (SCs ctrl). mOSKM was detected exclusively in D0 bFF-iPSCs mOSKM, and *PAX7* was specific to SCs ctrl. Technical duplicates were performed for each time point, and the results are presented as the mean ± SD to ensure accurate representation. Bars labeled with different letters are different from each other (*p* < 0.05).

Upon plating onto Matrigel with SKM01 media, the bFF-iPSCs mOSKM line displayed morphological changes, adopting either a fibroblast-like appearance or irregular cytoplasmic boundaries. By day 4, no biPSC colonies were present, indicating the onset of differentiation. By day 8, elongated cells emerged alongside smaller, round cells resembling satellite cells (SCs) (38) (Figure 4A).

RT-qPCR analysis revealed the presence of *OCT4* and *PAX3* transcripts, while SOX2, *NANOG*, and *mOSKM* were downregulated by day 8. Nevertheless, no *PAX7* expression was observed, which was present in the SC control (Figure 4B).

**Figure 5:**
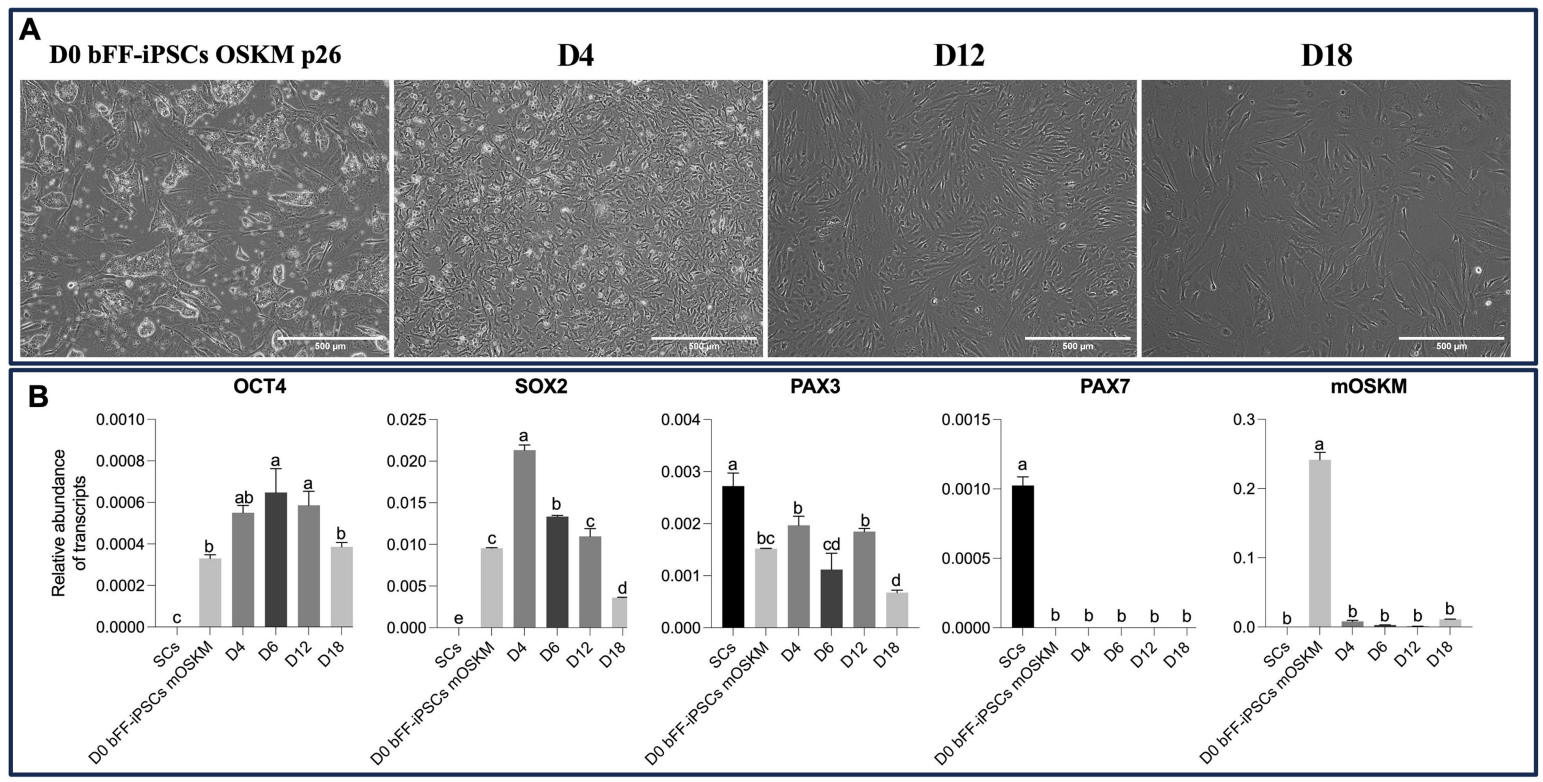
Impact of long-term culture on pluripotent and myogenic markers in bFF-iPSCs mOSKM. **(A)** Morphological changes of bFF-iPSCs mOSKM at passage 26 following 18 days of culture in SKM01 media. By day 4, cells began to adopt a fibroblast-like morphology. By days 12 and 18, cells maintained a fusiform shape and showed increased elongation (scale bar: 1000 μm at D0 and 500 μm at D4, D12, and D18). **(B)** Relative transcript abundance of pluripotency (*OCT4* and *SOX2*) and early myogenic genes (*PAX3* and *PAX7*) at different time points of differentiation (D0, D4, D12, and D18), compared to bovine primary satellite cells (SCs-Ctrl). *OCT4* and *SOX2* expressions decreased over time, while PAX3 levels progressively increased. However, no *PAX7* expression was detected during differentiation, and mOSKM expression decreased throughout the protocol. Technical duplicates were performed for each time point, and the results are presented as the mean ± SD to ensure accurate representation. Bars labeled with different letters are different from each other (*p* < 0.05).

Initial results showed that the bFF-iPSCs mOSKM appeared to start *in vitro* differentiation into SCs; however, 8 days in culture were not enough to further progress in the differentiation process. Therefore, a “long-term” condition was implemented using SKM01 media for 18 days on Matrigel. Throughout this longer culture period, the cells underwent morphological changes similar to those observed in previous reports (19). However, by the end of the protocol, most cells exhibited a fibroblast-like morphology (Figure 5A). Additionally, during the initial days of the differentiation protocol, *OCT4* and *SOX2* expression were clearly detected, suggesting that the pluripotent state was being maintained. Subsequently, both endogenous and *mOSKM* abundance were diminished over the course of the protocol. Although *PAX3* was initially detected at high levels, its expression unexpectedly decreased after day 4, and no *PAX7* expression was detected (Figure 5B).

#### 3.3.2 Differentiation of bFF-iPSCs derived from pMK episomal reprogramming into SCs

**Figure 6:**
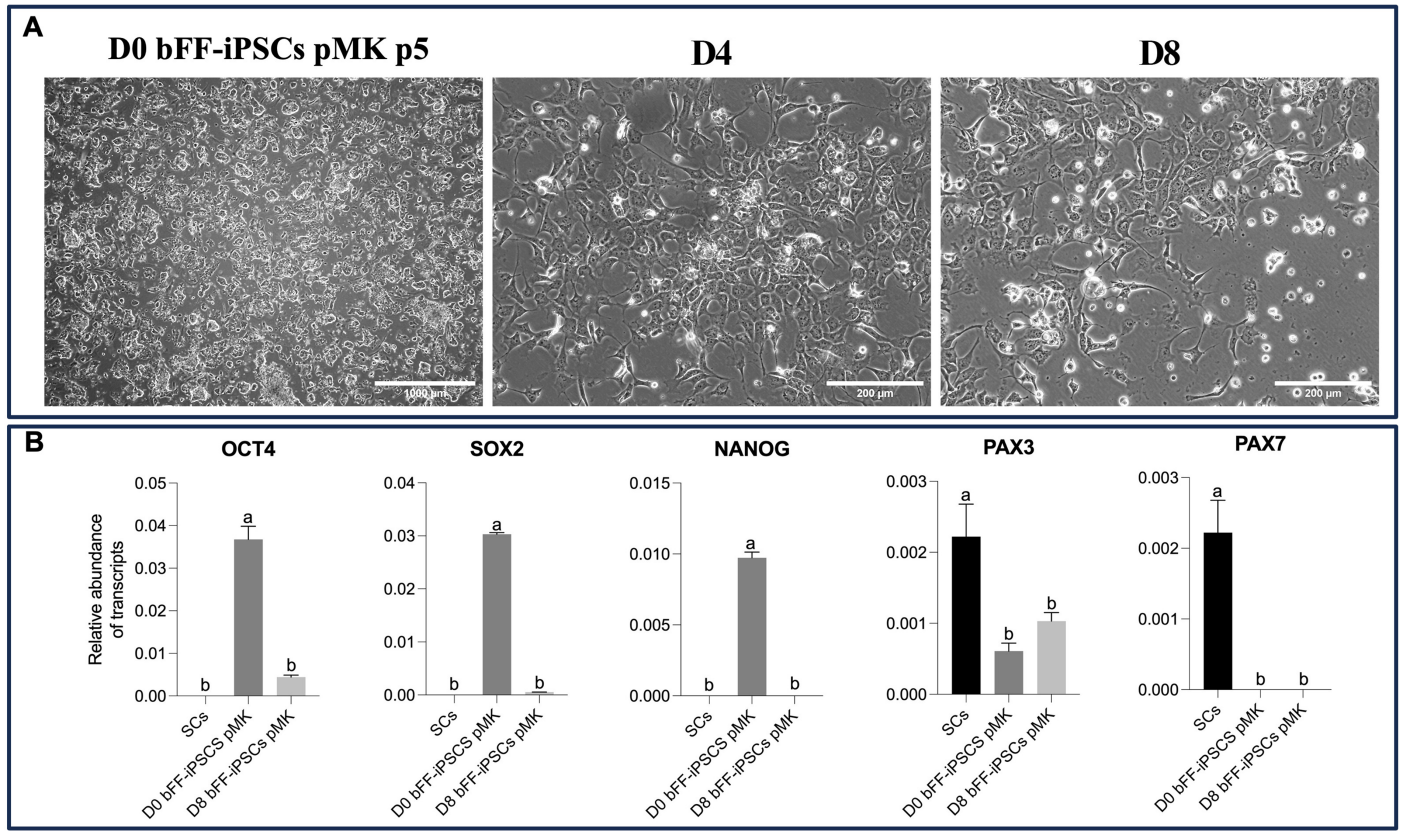
Impact of long-term culture on pluripotent and myogenic markers in bFF-iPSCs pMK **(A)** Morphological changes of bFF-iPSCs pMK at passage 5 following 8 days of culture in SKM01 media. At day 0, cells exhibited a compact, colony-like morphology characteristic of pluripotent stem cells. By days 4 and 8, cells displayed elongated edges, with some maintaining a large nucleus-cytoplasm ratio (scale bar: 1000 μm at D0 and 200 μm at D4, D8). **(B)** Relative transcript abundance of pluripotency markers (*OCT4* and *SOX2*) and early myogenic markers (*PAX3* and *PAX7*) in bFF-iPSCs pMK at different time points (D0, D4, and D8), compared to bovine primary satellite cells (SCs-Ctrl). As expected, pluripotency genes (*OCT4* and *SOX2*) were downregulated, and early myogenic marker PAX3 increased, indicating progression toward differentiation. However, no PAX7 expression was detected. Technical duplicates were performed for each time point, and the results are presented as the mean ± SD to ensure accurate representation. Bars labeled with different letters are different from each other (*p* < 0.05).

After being plated onto Matrigel with SKM01 media, the bFF-iPSCs pMK line rapidly began to change its morphology. No biPSC colonies were observed, and a few cells exhibited a fibroblast-like morphology, while others appeared more round and smaller, resembling SCs (38). This morphology was maintained until day 8 (Figure 6A). As expected, the abundance of *OCT4* and *SOX2* transcripts was found to be lower, while the *PAX3* transcript was increased at D8. However, no *PAX7* transcript was detected in the biPSCs or in D8 (Figure 6B).

**Figure 7:**
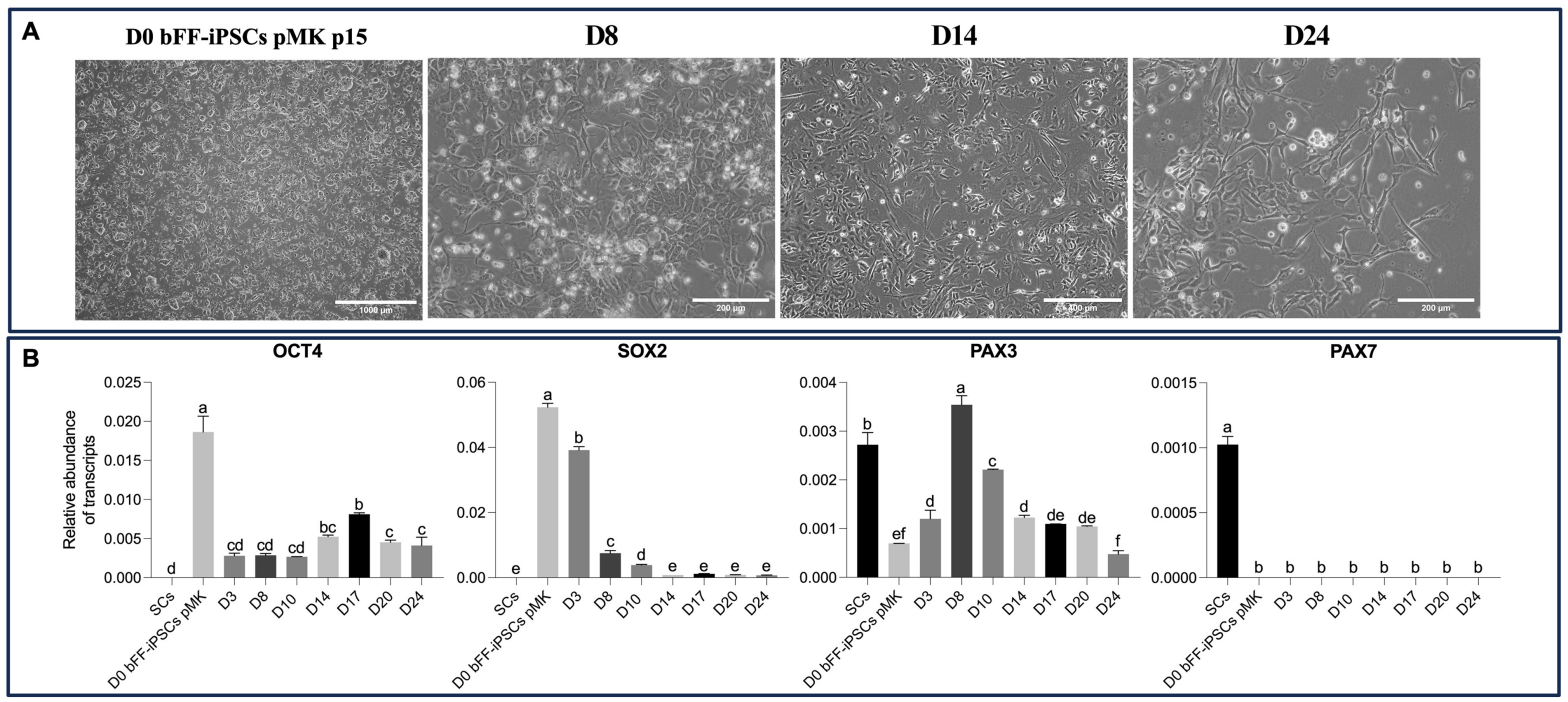
Morphological and gene expression changes during long-term differentiation of bFF-iPSCs pMK. **(A)** bFF-iPSCs pMK at passage 15 and their morphology differentiation after the long-term cultured with SKM01 media for 24 days. At D0, cells exhibit a compact and colony-like morphology. By day 8, cells began to display a more dispersed and elongated morphology. By day 14, further elongation was observed, with some cells adopting a spindle-shaped appearance. By day 24, an increased number of elongated cells was present (Scale bar: 1000 μm at D0, 400 μm at D8 and D14, and 400 μm at D24). **(B)** Relative transcript abundance of pluripotency markers (*OCT4* and *SOX2*) and early myogenic markers (*PAX3* and *PAX7*) in bFF-iPSCs pMK at different time points (D0, D8, D14, D17, and D24), compared to bovine primary satellite cells (SCs-Ctrl). A progressive downregulation of pluripotency genes (*OCT4* and SOX2) was observed, along with a fluctuating expression pattern of *PAX3*, which stabilized after day 14. No *PAX7* expression was detected. Technical duplicates were performed for each time point, and the results are presented as the mean ± SD to ensure accurate representation. Bars labeled with different letters are different from each other (*p* < 0.05).

Based on these findings, a long-term culture protocol was established using SKM01 media on Matrigel for 24 days. Throughout the culture period, the cells underwent the expected morphological changes. However, by the end of the protocol, most cells exhibited fibroblast-like morphology (Figure 7A). *OCT4* expression was detected throughout the protocol, although it decreased following differentiation. Similarly, *SOX2* expression was reduced over the 24-day differentiation period. *PAX3* expression initially increased but began to decrease after day 8. *PAX7* was undetectable in all biPSCs but was present in the primary satellite cells (Figure 7B).

In both attempts to differentiate bFF-iPSCs pMK into SCs, the cells seemed to initiate differentiation with increased levels of *PAX3* and a lower abundance of the pluripotent transcripts. However, after day 8, *PAX3* expression was progressively reduced and expression of the pluripotency marker *OCT4* was increased.

To determine whether the protocol could be improved, a combination of protocols was implemented, as described below. In summary, SKM01 media was utilized until day 8, after which it was replaced with the media previously reported by Chal et al. (15).

**Figure 8:**
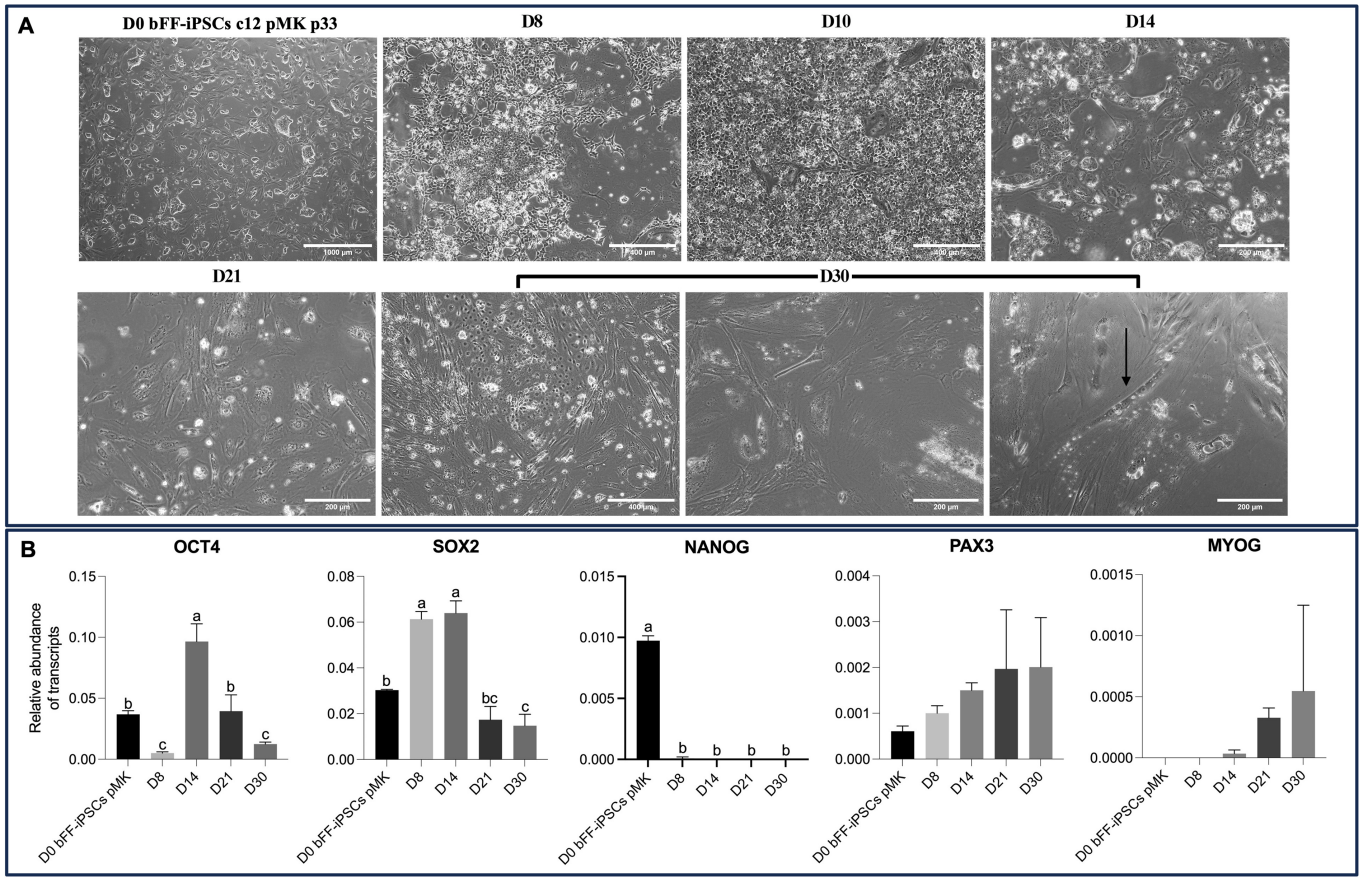
Extended culture effects on morphology and gene expression of bFF-iPSCs pMK. **(A)** Morphological changes of bFF-iPSCs pMK at passage 33 following 30 days of differentiation, showing characteristics indicative of myogenesis. By day 30, various cell types were observed, including cells with elongated, multinucleated morphology typical of myotubes (arrow) (scale bar: 1000 μm at D0, 400 μm at D8, D10, and D30-1 or 200 μm at D14, D21, D30-2, and D30-3). The arrow highlights a multinucleated cell at day 30. **(B)** Transcript levels of pluripotency markers (*OCT4* and *SOX2*) and myogenesis markers (*PAX3*, *PAX7*, and *MYOG*) at different time points during the differentiation protocol. *OCT4* and *SOX2* expressions were significantly reduced by day 30, while *PAX3* expression remained elevated, comparable to the primary bovine satellite cell control. *MYOG* expression was strongly induced after day 21. Notably, *PAX7* expression was undetectable at all time points. Technical triplicates were performed for each time point, and the results are presented as the mean ± SD to ensure accurate representation. Bars labeled with different letters are different from each other (*p* < 0.05).

By Day 30, various cell types were observed. Among these various cells, cells presenting putative myotubes with elongated morphology and multinuclear features were detected (Figure 8A). Concomitantly, expression of *OCT4* and *SOX2* was remarkably reduced, while PAX3 expression was maintained at comparable levels to the primary bovine satellite cell control sample. *PAX7* expression, however, was still not detectable. Interestingly, *MYOG* expression was strongly induced in biPSCs differentiation cultures after day 21 (Figure 8B). MYOG is typically found in myocytes and myotubes and stimulates further differentiation of SCs into these cell lineages (15, 39).

## 4 Discussion

The bFFs were successfully reprogrammed after nucleofection of episomal plasmids, and the clonal line bFF-iPSCs pMK demonstrated rapid proliferation, formed colonies with well-defined borders, large nuclei, exhibited AP activity, and transcripts and markers associated with pluripotency. Nevertheless, the bFF-iPSCs pMK line at passage 17 showed the presence of the episomal plasmids, which could indicate that either the episomal plasmids were not lost during cell divisions or that they may have integrated into the cell’s genome, similar to all bovine iPSCs generated to date (22, 23, 25, 40–44).

Herein, bovine iPSCs generated via episomal methodology were maintained in the media optimized by Zhao et al. (45), where biPSCs generated through PiggyBac Dox-inducible transposon and isolated bovine ESCs were successfully maintained *in vitro* for 63 passages. Additional supplementation was carried out using 10 ng/μl bFGF, which was found to be critical for self-renewal and proliferation through MEK/ERK and PI3K signaling pathways and helped to prevent differentiation (46). A lower concentration of antibiotics was used, and no Leukemia Inhibitory Factor (LIF) was added to the media once previous studies demonstrated that bFGF is more efficient in promoting the acquisition of pluripotency (22).

Differentiation of pluripotent stem cells into muscle cells through *in vitro* myogenesis has been well studied in humans, while it remains not fully elucidated in large animals such as pigs and cattle. Studies have reported that the cells need to undergo morphological changes and exhibit a higher abundance of PAX3, which plays a crucial role during early myogenesis (3, 35, 47). PAX7 expression is commonly used to identify SCs, which are essential for tissue regeneration and development (11, 15, 35, 38, 48). In our study, both PAX3 and PAX7 primers were validated using SCs as controls (11).

The biPSCs lines described herein showed to be suitable for the myogenesis protocol. In the initial attempt to differentiate both lines, a commercial protocol was employed, and the cells were maintained in culture for 8 days. By day 8, the bFF-iPSCs mOSKM lentiviral-derived line exhibited *OCT4* and *PAX3* expressions, lower levels of *SOX2*, and importantly, silencing of *mOSKM*; however, no *PAX7* transcripts were detected. After initial differentiation of the bFF-iPSCs pMK episomal-derived line, all pluripotent transcripts were detected in a lower magnitude, as expected. Previous reports using human PSCs by day 10 using this commercial media (19) indicated approximately only 20% of the population being satellite cells expressing the PAX7 marker (49).

During the extended culture period employed herein, both cell lines initially exhibited higher levels of *PAX3* and reduced abundance of pluripotent transcripts, as expected (19, 32). Interestingly, *PAX7* was not detected at any of the time points analyzed, which may suggest that the long-term culture with SKM01 media did not sufficiently induce further differentiation, or else, the exact window of PAX7 expression was not evaluated.

Hence, we next used a combined protocol with the use of SKM01 media for initial differentiation until day 8, when the bFF-iPSCs pMK episomal-derived line exhibited a higher relative abundance of *PAX3* transcripts. This was followed by replacing the culture media with the DK-HIFL media, as described by Chal et al. (15), which was used after the emergence of a higher abundance of the *PAX3* transcripts in the cell lines population.

The DK-HIFL media is supplemented with LDN-193189, hepatocyte growth factor (HGF), insulin growth factor 1 (IGF-1), and basic fibroblast growth factor (bFGF), all of which play an important role in myogenesis differentiation. LDN-193189 inhibits BMP signaling, a crucial function considering BMP’s established role in promoting the development of lateral plate mesoderm derivatives, including hematopoietic and cardiovascular cell types, both *in vivo* and *in vitro* (50–52). Furthermore, HGF, supplemented in the media, is produced by the lateral plate mesoderm and is essential for proper myoblast migration (53–55). The insulin (IGF-1) signaling, in collaboration with Wnt signaling, has been shown to promote myogenesis and myoblast fusion (56, 57). Moreover, bFGF signaling plays a role in promoting myoblast proliferation while impeding differentiation (58). Additionally, the modulation achieved in the pathways IGF-1, MAPK/Erk, TGF-*β*, and Wnt/β-catenin with the different cytokines used has shown to be of great importance during the human or mouse myogenesis (59–62); however, the details of these pathways in bovine remain not fully elucidated.

During the culture period, the cells exhibited a higher abundance of *OCT4* and *SOX2*, followed by a subsequent decrease until the end of the protocol. *PAX3* was found in higher levels and maintained its abundance throughout the culture period, in contrast to the results observed when using only the SKM01 media. No expression of *PAX7* was detected; however, the *MYOG* expression was found to be increased after day 21, correlating with the morphological changes observed in elongated cells with multinuclear characteristics typical of myotubes. These results are supported by the study of Zhu et al. (63) in porcine. Additionally, morphologies of other cell types could be observed, as described by Chal et al. (15) and Wada et al. (18).

## 5 Conclusion

In this study, we successfully generated biPSCs from fetal fibroblasts and utilized two biPSC lines to further investigate *in vitro* myogenesis. The optimization of a standardized human protocol using commercial media initially induced the differentiation of bFF-iPSC lines, with elevated PAX3 levels observed until day 8. Subsequently, the combination of commercial and lab-made media in long-term culture maintained *PAX3* abundance and facilitated the formation of multinucleated, elongated cells with *MYOG* transcripts persisting until the protocol’s conclusion. This achievement in the bovine model holds significant promise for advancing cultured meat production. In conclusion, ur findings mark progress in successfully deriving episomal bovine iPSCs and establishing a preliminary protocol for bovine *in vitro* myogenesis. These milestones facilitate translational studies in veterinary and pave the way for the development of innovative technologies in tissue engineering, such as alternative animal protein production. As the global demand for alternative protein sources grows, CM, produced through cellular agriculture and tissue engineering principles, presents a promising approach. As CM continues to evolve, addressing these challenges will be critical for realizing its commercial potential, offering a broad impact across the biotechnology and food sectors.

## Data Availability

The original contributions presented in the study are included in the article/Supplementary material, further inquiries can be directed to the corresponding author.

## Footnotes

^1^ www.graphpad.com
