# Supplementary material for: Generation of bovine iPSCs from fetal fibroblasts for in vitro myogenesis and cultured meat
Source: Front Nutr. 2025 May 16;12:1562981. doi: 10.3389/fnut.2025.1562981 (PMC12124125; doi:10.3389/fnut.2025.1562981)
Supplement: Supplementary file 1 [file Table_1.DOCX]

Generation of bovine iPSCs from fetal fibroblasts for in vitro myogenesis and cultured meat

Kaiana Recchia^1^, Methi Wahithinnakon^3^, Fabiana Fernandes Bressan^1,2^, Kristine Freude^3^*

^1^Surgery Department, Faculty of Veterinary Medicine and Animal Sciences, University of São Paulo, São Paulo 01001-010, SP, Brazil

^2^Department of Veterinary Medicine, Faculty of Animal Sciences and Food Engineering, University of São Paulo 13635-000, Pirassununga, SP, Brazil

^3^Department of Veterinary and Animal Sciences, Faculty of Health and Medical Sciences, University of Copenhagen, 1870 C Frederiksberg, Denmark

*** Correspondence:**

Corresponding Author

Kristine Freude kkf@sund.ku.dk

**Supplementary table 1.** Bovine specific primers used for pluripotency and myogenic characterization, and primers for mOSKM and oriP-pMaster K detection.

| **Target** | **Forward** | **Reverse** | **bp** |
| --- | --- | --- | --- |
| bGAPDH | TCCTGCCCGTTCGACAGATA | AAGGGGTCATTGATGGCGAC | 166 |
| bPPIA | CCGCGTCTCTTTTGAGCTGT | ACCCTGGCACATAAATCCCG | 139 |
| bOCT3/4 | CTGGAGAAAGACGTGGTCCG | GAGACCCAGCAGCCTCAAAA | 106 |
| bSOX2 | ATGGGCTCGGTGGTGAAGT | TGGTAGTGCTGGGACATGTGA | 178 |
| bNANOG | TGTGGAGGAGAGCACAGAGA | CCAGGTCTTCACCTGCTTGT | 187 |
| bPAX3 | CGCCACAAGATCGTGGAGAT | GTTGTCACCTGCTTGGGCT | 167 |
| bPAX7 | CTCCCTCTGAAGCGTAAGCA | GGGTAGTGGGTCCTCTCGAA | 95 |
| bMYOG | CGAGTGCCCCTTGAAGACAA | CCACAGACACCGACTTCCTC | 103 |
| mOSKM | ACGAGCCACAAGCTCACCTCT | GGCATTAAAGCAGCGTATCC | 221 |
| Orip-pMaster K | TTCCACGAGGGTAGTGAACC | TCGGGGGTGTTAGAGACAAC | 544 |

**
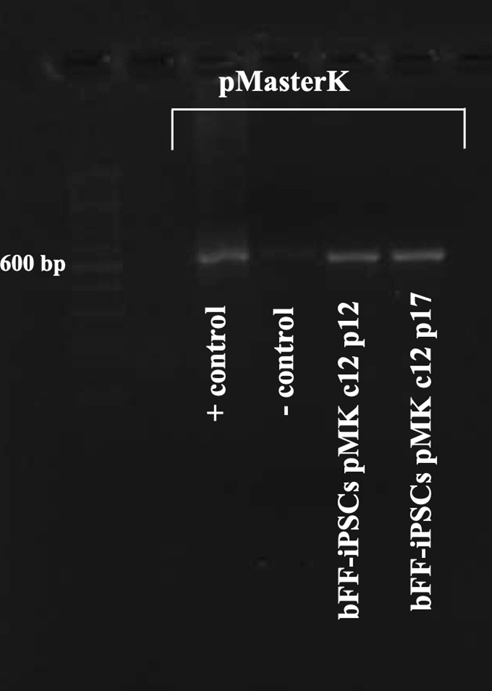
**

**Supplementary figure 1.** Detection of pMaster K plasmid on bFF-iPSCs pMK clonal line 12 at passage 17 (positive control: pMaster K plasmid, negative control: DNA from bovine fetal fibroblasts - bFF).
